# Destructive tsunami-like wave generated by surf beat over a coral reef during Typhoon Haiyan

**DOI:** 10.1038/ncomms8854

**Published:** 2015-08-06

**Authors:** Volker Roeber, Jeremy D. Bricker

**Affiliations:** 1 International Research Institute of Disaster Science, Tohoku University 468-1-E304 AzaAoba, Aramaki, Aoba-ku, Sendai 980-0845, Japan

## Abstract

Storm surges cause coastal inundation due to setup of the water surface resulting from atmospheric pressure, surface winds and breaking waves. Here we show that during Typhoon Haiyan, the setup generated by breaking waves near the fringing-reef-protected town of Hernani, the Philippines, oscillated with the incidence of large and small wave groups, and steepened into a tsunami-like wave that caused extensive damage and casualties. Though fringing reefs usually protect coastal communities from moderate storms, they can exacerbate flooding during strong events with energetic waves. Typical for reef-type bathymetries, a very short wave-breaking zone over the steep reef face facilitates the freeing of infragravity-period fluctuations (surf beat) with little energy loss. Since coastal flood planning relies on phase-averaged wave modelling, infragravity surges are not being accounted for. This highlights the necessity for a policy change and the adoption of phase-resolving wave models for hazard assessment in regions with fringing reefs.

## Abstract (web summary)

The failure of a broad fringing coral reef to protect the village of Hemani from a tsunami-like wave during Typhoon Haiyan came as a destructive surprise. Here, the authors present results from a phase-resolving wave model and show that the steep reef face facilitated the release of energetic infragravity waves.

## Graphical abstract

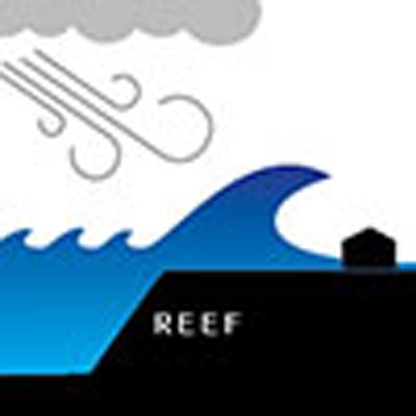

The town of Hernani is a fishing and agricultural community exposed to the open Pacific Ocean in Eastern Samar, in the Philippines (Fig. 1). The tsunami-like wave that struck the town during Typhoon Haiyan on 8 November 2013 (Fig. 2)^1^ astonished both villagers and disaster managers, as the coast near Hernani is sheltered by a broad fringing coral reef, which was expected to serve as a reliable wave defence^2^. During the authors' interviews of town residents as well as the survey conducted by Nobuoka *et al*.^3^, witnesses reported that three to four destructive bores struck the town, and that the duration of the flooding caused by each bore was on the order of minutes. Since storm-induced ocean waves generally have periods of <20 s, the nature of this event has remained a mystery.

Media have reported this event as a meteotsunami^4^ or as storm surge^5^, albeit storm surge refers to the elevated still water level due to barometric-, wind- and breaking-wave-induced setup, but not to wave-induced oscillations^6^. A meteotsunami can form when the forward propagation speed of a storm is close to the free long wave speed of the water body over which it travels (Proudman resonance)^7^,^8^. The translation speed of Typhoon Haiyan was at most 14 m s^−1^ (refs 9, 10) but the propagation speed of a free long wave is *c*=(*gh*)^1/2^, where *g* is the acceleration due to gravity and *h* is the local water depth. This would have required a water depth on the order of 20 m or less for the storm to have been able to keep up with the free long wave generated. However, the water depth offshore of Hernani quickly drops to kilometres (Fig. 1), precluding the possibility of a meteotsunami at this location.

On the basis of the authors' own interviews on site, local residents believed that the typhoon triggered a landslide, which in turn caused a near-field tsunami. However, no tsunamigenic earthquake or landslide activity was reported. Furthermore, a similar tsunami-like flood was reported to have occurred during the 12 October 1897 typhoon^11^, underscoring the likelihood that the bore was a direct consequence of the typhoon, and not a tsunami that happened to occur simultaneously.

The grave significance of the damage in Hernani is that the mechanism that caused the destruction is unaccounted for in coastal hazard assessment, evacuation planning or structural building codes. In the deep water offshore of Hernani, wind-driven setup only grew to a few decimetres, similar in size to the setup caused by the barometric pressure drop^12^. This is in contrast to the situation of the area around Tacloban city (Fig. 1), which is located at the end of a shallow bay with a maximum water depth of 50 m. The strong winds from Typhoon Haiyan, together with a local basin and shelf seiche, pushed enough water onshore to cause up to 6 m of storm surge to flood the city^13^. In addition, the local wind-generated waves on top of this surge also destroyed buildings along the shoreline^12^. Storm surge and damage of the type seen in Tacloban was predicted with great accuracy^14^, as similar wind-driven surges have been studied for decades, with some outstanding examples being Hurricane Katrina in the United States, the Bhola cyclone in Bangladesh and the Ise-Bay typhoon in Japan. Each of these events affected low-lying land at the head of a shallow bay, similar to the setting of Tacloban. Wind-driven storm surge is well understood because of its frequent occurrence and large geographic extent. Furthermore, the physics of wind-driven storm surge is a simple balance between pressure gradient and parameterized wind stress, allowing it to be calculated with depth-averaged hydrostatic approximations of the Navier–Stokes equations. The physics responsible for damage in Hernani, however, are different.

Here results show that a numerical storm surge model coupled with a phase-averaged wave model cannot reproduce the destructive long-period wave that struck the town. A phase-resolving wave model, however, successfully captures this phenomenon because it resolves individual waves, the incidence of wave groups and the steepening of the resulting surf beat into a bore. The study illustrates that the abrupt wave-breaking process over the steep reef face that freed the initially bound underlying group wave energy was the driving factor of the destructive tsunami-like bore. Resonant amplification over the reef flat, which is a common process associated with tsunami waves^15^, did not contribute significantly to the described wave phenomenon. Consequently, the wave would have been amplified if the incident wave group period had been closer to the reef's resonant period. Though Hernani's fringing reef protects the town from moderate storms, it exacerbated damage during Typhoon Haiyan therefore demonstrating that major storms bare the potential of locally generating tsunami-type flooding. This process is not being accounted for in disaster management plans or building codes. Phase-resolving wave models are suitable tools to account for this phenomenon in hazard planning, especially along reef-protected coasts around the world.

## Results

### Storm surge and phase-averaged wave model

With deep water offshore of Hernani, a parametric hurricane model (Methods) together with the Delft-3D/SWAN^16^,^17^ flow and phase-averaged spectral wave model calculates a combined wind- and pressure-driven surge of about 1.5 m above mean sea level (MSL)^12^. This modelling approach is the type used by the Federal Emergency Management Administration and the National Flood Insurance Program to determine the coastal hazard zone for flood insurance rate map assessment in the United States. The breaking-wave-induced radiation stress gradient induced an additional setup component of 2 m, resulting in a flow depth of 0.5 m and a flow speed up to 0.8 m s^−1^ (Fig. 3a) at the location of the house in Fig. 2, where the ground is 3 m above MSL. In addition, the model calculated short waves of 11 s peak period and 0.5 m significant wave height at this location. However, the computed surge was neither deep enough nor energetic enough to result in the flooding seen in Fig. 2, in which the flow depth and speed were around 2 m and 5 m s^−1^, respectively, and landward-directed flow duration at the house was a minute or longer^3^.

### Background on tsunami-like surf beat

Previously, Nakaza and Hino^18^ observed a similar wave phenomenon in Okinawa, which they investigated with a small-scale laboratory experiment. Nwogu and Demirbilek^19^ utilized a phase-resolving Boussinesq wave model to analyse a similar set of laboratory experiments. The fundamental dynamics involved in this phenomenon are multiple. The presence of alternating large and small incident wave groups (also called sets) causes the wave setup on the beach to vary in time, resulting in surf beat with an infragravity period on the order of minutes^20^,^21^. The minimum wave envelope (group) return period^22^ is a function of the shape of the offshore wave spectrum (Methods, equations (1) and (2)). In the frequency domain, surf beat energy may overlap with the natural quarter-wave oscillator frequency of the reef (Methods, equation (3)), and thus can amplify due to resonance. As the surf beat shoals, it can steepen and form a bore^18^,^23^ (Methods). Individual incoming ocean waves also propagate on top of the wave setup, interact with it and run-up on land as shorter bores.

### Phase-resolving wave model

To recreate the tsunami-like wave that struck Hernani, the offshore SWAN wave spectrum was input to the two-dimensional (plan view) Boussinesq-type phase-resolving wave model BOSZ^24^,^25^ (Methods). Figure 3b shows water level and flow speed time series in close agreement with the flow at the house in Fig. 2. Figure 4b shows the spatial extent of the 1-m high bore that strikes the house at *t*=625 s in Fig. 3b. In Fig. 3b, infragravity-period surges with steep bore-type fronts strike the house at model time 420 and 625 s. The interval between these bores is close to the theoretical return period of incident wave groups of 230 s evaluated from the offshore SWAN wave spectrum (Methods). The reef's resonant period, however, is ∼480 s (Methods) and therefore does not significantly amplify the peak energy of the surf beat. In addition to infragravity-period surf beat, individual gravity waves propagate atop the water surface.

### Wave group energy versus reef resonant amplification

**Figure 1:**
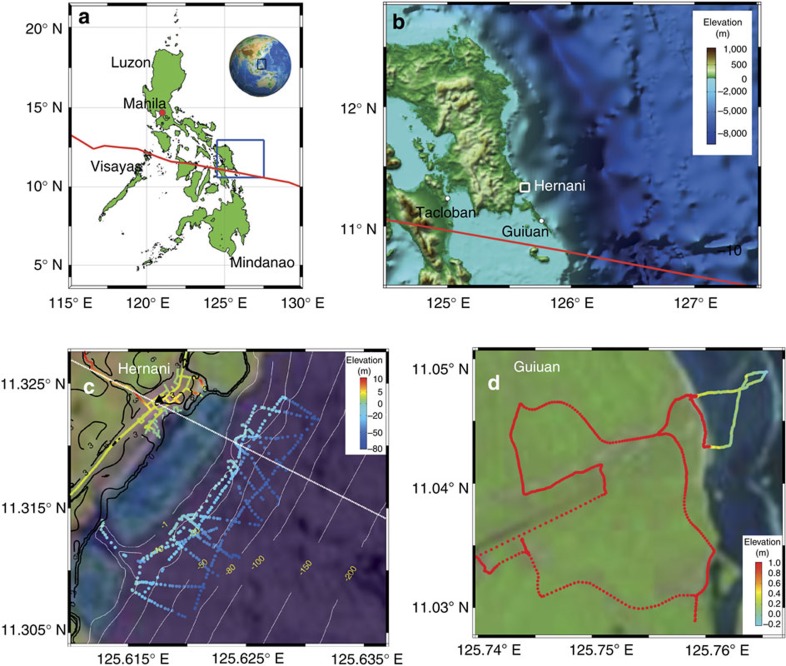
Topography and bathymetry of Hernani. (**a**) World locator map^49^ (inset) and locator map within the Philippines. (**b**) Ocean depth^34^ near Leyte and Samar Islands. The red line is the storm track of Typhoon Haiyan^9^. (**c**) Map of measured topography and bathymetry in Hernani (Methods). The white dotted line indicates the transect used in the one-dimensional model. The black triangle is the location of the house seen in Fig. 2. (**c**) Map of reef topography survey in Guiuan. Data available from the U.S. Geological Survey.

**Figure 2:**
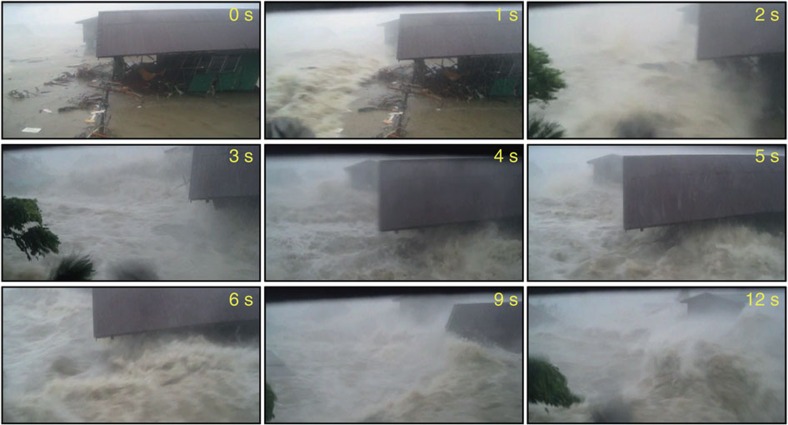
Frames from the video of Gensis^1^ showing a house being swept away by an infragravity-period wave. Onshore-directed flow lasted at least 20 s (the duration of the film). The base of the house is located 3 m above MSL. Images used with permission of Plan International.

To determine the relative contribution of offshore wave groups and reef resonance to amplification of the surf beat, BOSZ was evaluated in one-dimension along the transect shown in Fig. 5. For the actual bathymetry (‘reef') case, most of the water level variance on the beach is at *f*=0.0035 Hz (frequency *f* is the inverse of period *T*) as illustrated in Fig. 6. This is close to the theoretical wave group return frequency of *f*=0.004 Hz. The hypothetical case of ‘no reef' in Fig. 6 represents the bathymetry of the channel surveyed in Fig. 1, where freshwater river outflow prevented the reef from growing. For this ‘no-reef' case, a peak in variance exists near the wave group return frequency, but it is much smaller than in the real reef case. For the hypothetical case of the town's seawall being infinitely high (‘reef high wall' case in Fig. 6), another peak is present at *f*=0.002 Hz, which corresponds to the reef's resonant frequency. Resonance has a much larger effect here than in the real bathymetry case because the high seawall limits the domain to the exact size of the quarter-wave oscillator, whereas in the real reef case the domain is lengthened by the run-up of the surf beat on land. The case of a steep ‘drop-off' immediately offshore of the beach has a peak at the wave group frequency of 0.004 Hz of slightly larger magnitude than that of the ‘reef'. Since the reef face bathymetry is identical for both ‘reef' and ‘drop-off' cases, the similarity of the wave spectra between these two scenarios indicates that the mechanism responsible for the tsunami-like wave of Fig. 2 was mostly a result of the surf beat energy released through the abrupt wave-breaking process over the steep reef face. Resonant amplification of the surf beat over the reef flat played a secondary role. In contrast to the previous scenarios, the ‘no-reef' case has a gradual wave-breaking zone, resulting in less energy available for the transfer of energy from gravity to infragravity frequencies (Methods).

Interestingly, the wave that struck Hernani was not a worst-case scenario: if a similar storm to Typhoon Haiyan was to strike a site fronted by a narrower reef with an equally steep reef face, resonance over the reef would become significant, with the potential of forming an even larger surf beat and subsequently more intense flooding. This is illustrated in Fig. 6 by the hypothetical case of a reef with only half the cross-shore length of the Hernani reef. The resonant frequency of this ‘half reef' is 0.0042 Hz, which is close to the wave group return frequency and results in stronger amplification of the group wave energy compared with the ‘reef' case.

### Damage from large storms exacerbated by the reef

The effect of the presence of the reef on damage onshore was further investigated using one-dimensional BOSZ simulations. Storms of various intensities were modelled by scaling down the central pressure and maximum wind speed of Typhoon Haiyan (Fig. 7a). For each hypothetical storm, a drag law^26^ (Methods) approximates the hydrodynamic force *F* on buildings near the beach. Figure 7b–e shows the spectra of *F* (proportional to *hu*^2^, where *h* is water depth and *u* is flow speed) on the beach for the ‘reef' and ‘no-reef' cases. For storms of up to about 50% the strength of Typhoon Haiyan, the reef protects the shore from incident waves. During larger storms, however, it elevates the potential for damage due to an increasing hydrodynamic force in the infragravity frequency band.

## Discussion

The tsunami-like wave that struck Hernani was the result of abrupt breaking of waves over the steep reef face generating an energetic surf beat. Had the surf beat been in resonance with the reef flat^15^,^27^, the wave would have been even larger. Results show that at this site, the reef protects the shoreline during moderate storms, but can exacerbate damage during strong storms like Haiyan. This phenomenon was successfully reproduced by a phase-resolving wave model, indicating the necessity for adoption of such models in coastal hazard assessment for areas around the world that face a fringing reef-type bathymetry. Since the outlined tsunami-type flooding originated mainly from the energetic storm waves, the potential of such a destructive wave is not limited to tropical and subtropical areas with coral reefs. Alternately, sites in the path of extra-tropical storms can be exposed to similar scenarios if the local bathymetry favours the abrupt release of surf beat, which in turn can freely propagate onshore.

## Methods

### Field surveys

Land surface and coral reef elevations were measured from 25 to 27 May 2014 via walking and driving surveys with two Ashtech ProMark 100 differential global positioning system (DGPS) units, in base/rover differential configuration, and post-processed for vertical accuracy with GNSS Solutions software. The water depth seaward of the coral reef was measured from a hired fishing canoe, using a Hondex PS-7 handheld digital sounder, which has an operational range of up to 80 m. Sounder readings were photographed with a Canon PowerShot D20 global positioning system (GPS) camera to record the geographic location of each measurement. All elevation and depth measurements were referenced to MSL using the TPXO global tide model^28^.

Since the elevation of the coral reef flat in Hernani was not measured during the 25–26 May field campaign in Hernani, the reef flat elevation of a similar reef stretch in Guiuan (35 km Southeast of Hernani; Fig. 1d) was measured during low tide on 27 May, with the same DGPS base/rover configuration as described above, and corrected to local MSL using TPXO^28^. The measured reef flat elevation varied from 10 cm above MSL to about 10 cm below MSL, though many of the deeply ponded areas on the reef flat were avoided during the survey. Therefore, for the storm surge modelling to follow, the reef flat in Hernani was assumed to have an elevation of 5 cm below MSL in the area surrounded by the topography and bathymetry measurements show in Fig. 1.

### Parametric hurricane model

The behaviour of Typhoon Haiyan was hindcast using typhoon track data from the Japan Meteorological Agency^9^, which was input into Holland's parametric hurricane model^29^ for air-pressure field estimation, followed by the moving-typhoon model of Fujii and Mitsuda^30^ as described in Veltcheva and Kawaii^31^ for estimation of the wind field. Since the typhoon track data lacked information on radius to maximum wind, the relation of Quiring^32^ was applied^12^. The hindcast pressure and wind fields were input into Delft-3D^16^ and SWAN^17^, a combined hydrodynamic and phase-averaged wave model package to hindcast the still water level and significant wave heights induced by the typhoon. An important adjustment to the default SWAN setup was the use of an air–sea drag coefficient limiter of 0.003 to prevent unphysical wave growth^33^.

### Delft-3D/SWAN storm surge and phase-averaged wave model

The Delft-3D/SWAN model was run using domain decomposition. The large domain (125°<East longitude<130°, 10°<North latitude<15°) had a resolution of 0.01°(about 1 km), and the small domain (125.59°<East longitude<125.66°, 11.29°<North latitude<11.36°) had a resolution of 0.0004°(about 40 m). Coarse bathymetry data was taken from GEBCO^34^ and topography from SRTM^35^. Within the region of the field survey of Fig. 1c, the bathymetry and topography were interpolated from surveyed data. The Global Tide database TPXO^28^ provided the tidal boundary condition to the hydrodynamic model. Bed roughness was parameterized with a uniform Manning's *n* of 0.035 s m^−1/3^ (ref. 36). The Delft-3D model was run with a uniform time step of 6 s, while SWAN was run in stationary mode at 15-min intervals (a test was run in non-stationary mode, but results did not differ noticeably from stationary mode).

SWAN output was produced for various storm intensities to quantify the effect of the overall wave energy on the formation of the bore that struck Hernani. In Fig. 7a, the SWAN model result from Typhoon Haiyan^9^ is labelled the 100% intensity storm. The hypothetical 80% storm was determined by using a storm track with 80% of the pressure drop in the eye and 80% of the maximum wind speed of the Typhoon Haiyan track data, then re-evaluating the radius to maximum winds, the parametric hurricane model and the Delft-3D/SWAN model. The procedure was repeated for the 60 and 40% storms.

### BOSZ Boussinesq-type wave model

To generate the input wave time series for the phase-resolving BOSZ wave model^24^,^25^, SWAN directional wave spectra output were extracted in 150 m water depth offshore of Hernani. The SWAN spectra are composed of 25 logarithmically spaced frequency bins between 0.04 and 0.3 Hz and 72 directional increments of 5° each. Since SWAN does not provide the phase angles between the spectral components, the common approach^37^ is to treat each spectral component as a linear wave with a random initial phase. However, the discreteness of wave components leads to recycling of the superposed input time series and therefore to artificial wave groups with a return period of 1/d*f*. To avoid any artificial wave group generation, the SWAN spectra were resampled to a logarithmic spacing with a smallest interval of d*f*=1/3,600 s, which excludes repetitions of the input signal over the 1-h model run. Furthermore, to ensure quality of the BOSZ results, the high frequency tail of the SWAN spectra were truncated at 0.13 Hz and proportionally redistributed over the remaining frequency bins according to the constraints of the dispersion approximations in the model's governing equations, as described in Li *et al*.^38^.

The wavemaker source function used in BOSZ^24^,^25^ is built from those of Wei *et al*.^39^ and Schäffer and Sørensen^40^, and described in Li *et al*.^38^. To ensure uniform wave energy distribution over the portion of the BOSZ two-dimensional computational domain of uniform 150 m water depth, all three open ocean-facing domain boundaries contain wave-generating source functions. Wave shadowing due to the directionality of the spectral components is only visible in very limited regions along the Northern and Southern boundary (Fig. 4a). Therefore, the overall results of this study are not affected. For the one-dimensional analyses, the components from the two-dimensional wave spectrum are collapsed into a one-directional distribution of the wave energy over all frequency bins.

**Figure 3:**
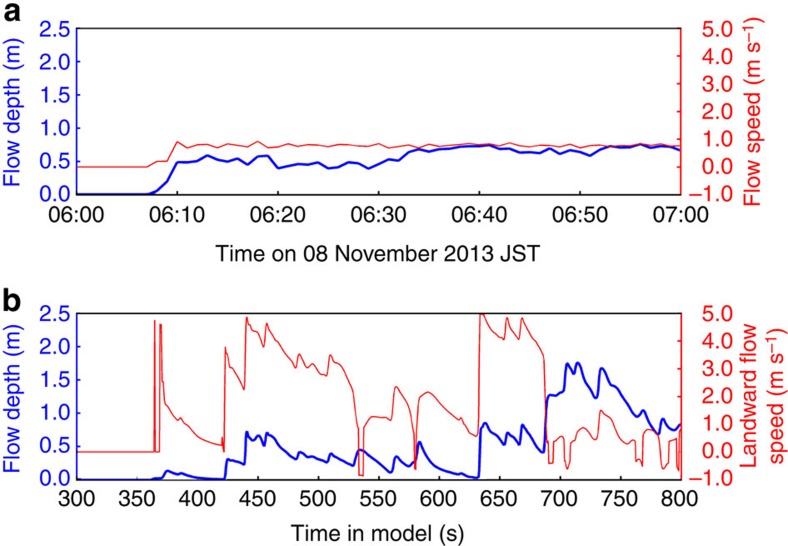
Computed wave time series and spectra illustrating the physics responsible for the wave of Fig. 2a. (**a**) Time series of water surface elevation and flow speed from the Delft-3D/SWAN model at the location of the house in Fig. 2b. (**b**) Time series of water surface elevation and flow velocity (positive for landward flow) from the two-dimensional BOSZ model at the location of the house in Fig. 2.

**Figure 4:**
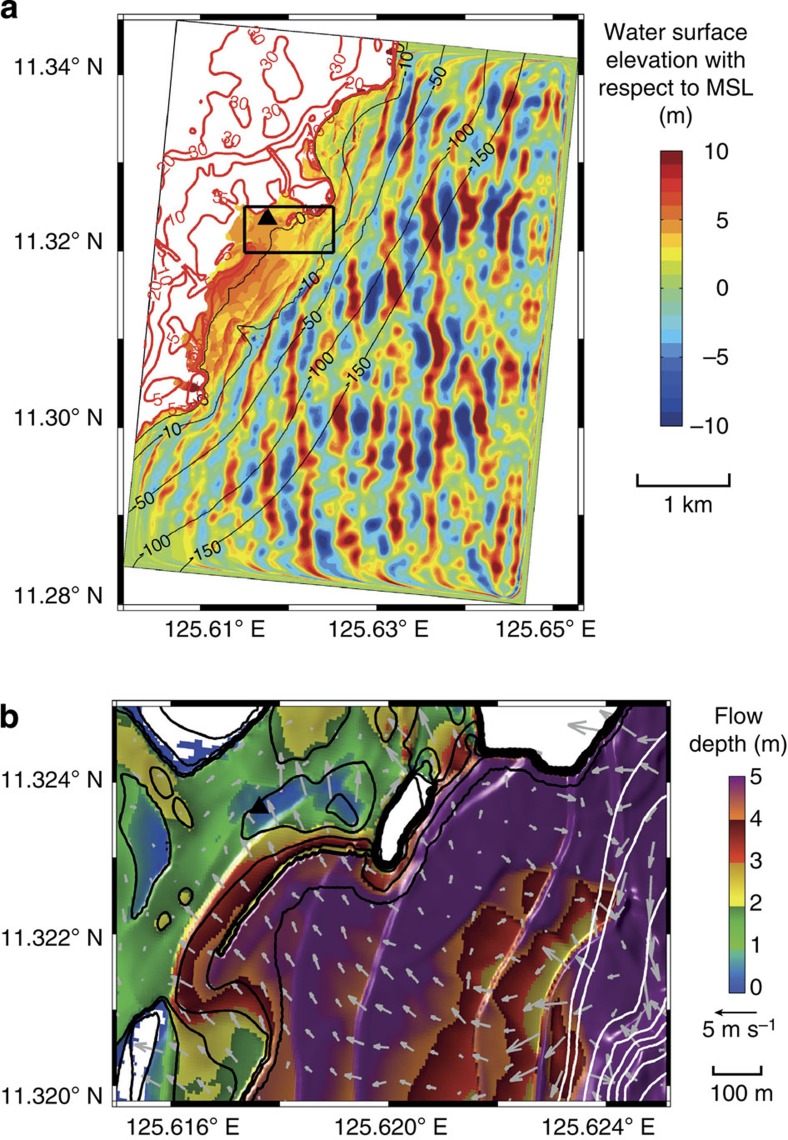
Flow depth throughout Hernani when a bore strikes the house of Fig. 2. (**a**) Snapshot at *t*=625 s of the two-dimensional BOSZ simulation. The black triangle indicates the location of the house in Fig. 2. Black and red lines are bathymetric and topographic contours, respectively, in metres above MSL. Wavemakers are located 290 m inside the domain from the South, East and North offshore boundaries. Sponge layers to absorb short-period oscillations are placed between the wavemakers and the boundary walls. The computed domain is 5 km long in the East–West direction and 6.9 m long in the North–South direction. An animation of this figure is available as Supplementary Movie 1. (**b**) Close-up on the reef and town. White lines are sea bottom elevation contours beginning at MSL and continuing downward at 5-m intervals. Black lines are land elevation contours beginning at 1 m above MSL and continuing upward at 1-m intervals to 10 m above MSL (higher elevations omitted). Arrows are flow velocity vectors. An animation of this figure is available as Supplementary Movie 2.

**Figure 5:**
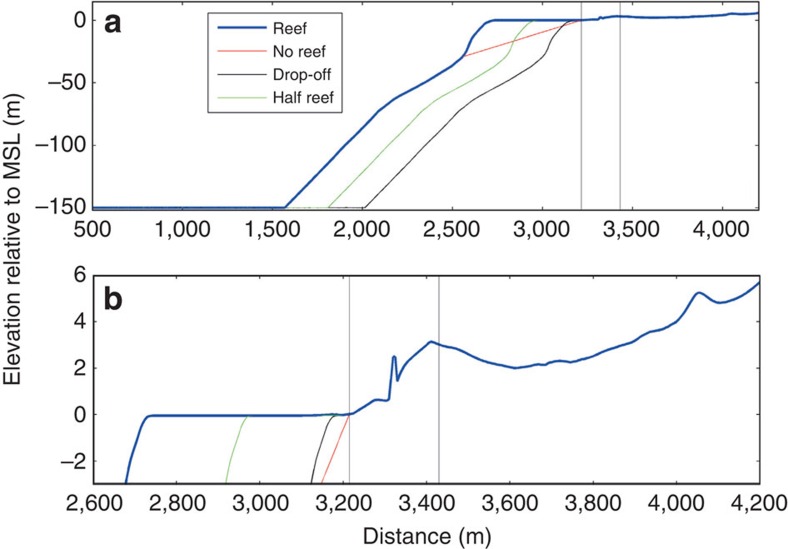
Bathymetry and topography of transect used in one-dimensional BOSZ computations. The abscissa indicates the distance *x* along the transect of Fig. 1c from offshore (Southeast) to onshore (Northwest). (**a**) Full domain. (**b**) Zoomed in topography of the reef and onshore region. The right-hand thin black line indicates the location of the house swept away by the tsunami as seen in Fig. 2 and the left-hand thin black line is the location of the beach. The kink between the two thin black lines is the town's seawall. The red, green and medium black lines are hypothetical bathymetries, while the thick blue line is actual bathymetry.

The resulting time series were used to evaluate the effects of surf beat and resonance over the reef in two dimensions (plan view) as well as along a one-dimensional transect passing through the location of the house seen washed away in Gensis' film^1^. The two-dimensional BOSZ model run was based on a grid with a uniform 5 m × 5 m resolution over a domain extending 5 km in the East–West direction, 6.9 km North–South, and rotated 5° clockwise to allow wave input centred around the peak direction as computed by SWAN (Fig. 4a). Owing to constraints in computational resources, only one two-dimensional computation was run for the results shown in Fig. 3b. Other cases were calculated with one-dimensional bathymetries based on a 5-m resolution (Fig. 5). For each bathymetry, 10 different one-dimensional BOSZ runs were evaluated, each with a unique random phase seed. Ensemble averages over these 10 runs for each bathymetry then lead to robust frequency spectra. Manning's *n* of 0.035 s m^−1/3^ (ref. 36) was used uniformly and results of a sensitivity run with no friction were not significantly different.

### Validation of BOSZ Boussinesq-type wave model

As no measurements of actual wave heights are available from the site, the phase-resolving depth-resolving Volume-of-Fluid model OpenFOAM^41^, version 2.3.0, facilitates a redundant check of the validity of the conclusions reached with BOSZ. OpenFOAM was applied along the same one-dimensional transect with an identical input wave time series. The OpenFOAM model setup is described in detail in Bricker and Roeber^42^. Figure 8 shows a comparison between the results from BOSZ and OpenFOAM on both sides of the seawall shown in Fig. 5. The reasonable agreement between these two different types of phase-resolving wave models gives credence to the BOSZ results, in lieu of field measurements to compare with. In addition, BOSZ has been intensively validated with laboratory experiments conducted at the O.H. Hinsdale Wave Research Laboratory at Oregon State University that were specifically designed for wave propagation and bore formation over fringing reef-type bathymetries^43^. BOSZ and the data used to drive it are available via the contact on the authors' website at http://hydraulic.lab.irides.tohoku.ac.jp/.

### Detailed results from BOSZ wave model

The model results can be understood through comparison with analytical oscillation periods of the water surface. In the incident wave time series, the minimum wave group return period is given by equation (1) (ref. 22)


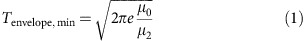


where *μ*_0_=*m*_0_ and *μ*_2_=*m*_2_−*m*_1_^2^/*m*_0_. The spectral moments *m*_*r*_ are given by equation (2)


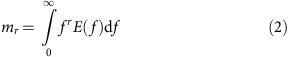


where *f* is the frequency (Hz), *E* is the spectral energy density (m^2^ Hz^−1^) and *r* is an integer. The actual wave group return period can be longer than *T*_envelope,min_. For Typhoon Haiyan *T*_envelope,min_ is 230 s, as represented by the 100% storm of Fig. 7a with a high frequency tail cutoff at 0.13 Hz. It should be noted that the cutoff position can slightly influence the group return period based on a shift in the spectral moments.

The resonant period of a quarter-wave oscillator such as a reef flat is given by equation (3)


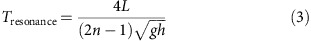


where *T*_resonance_ is the resonant period, *L* is the cross-shore length of the reef, *g* is the acceleration due to gravity, *h* is the mean water depth over the reef and *n* is a positive integer defining the amount of nodes. This type of oscillation consists of a node at the reef face and an antinode at the beach. Assuming *L*=750 m (the distance between the seawall and the 30-m bathymetric contour), *h*=4 m (the mean water depth over the reef) and *n*=1 (the fundamental period of oscillation), the reef resonant period is 480 s (corresponding to a frequency *f*=0.0021 Hz).

The period of a basin seiche, a numerical artifact of the closed model domain, is given by equation (4)


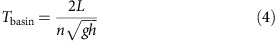


with the same variable definitions as in equation (3). This type of oscillation consists of an antinode at each side of the domain. For the one-dimensional transect of Fig. 5, the fundamental (*n*=1) seiche period is the time it takes a long wave to propagate from the left wall of the domain, reflect from the reef face (at *x*=2,700 m, which is the shoreward-most extent of this energy band in Fig. 9) and then propagate back to the left boundary. Numerically solving for the long wave speed in discrete cells across the variable-depth basin, this fundamental period is found to be 165 s (*f*=0.006 Hz).

Figure 9 shows that for the real reef bathymetry, incident waves from offshore break on the reef crest at a distance of *x*=2,700 m from the start of the transect. Landward of this location, energy in the gravity wave range is low, but a non-negligible amount of energy exists even beyond the seawall at *x*=3,314 m, indicating ocean waves propagate atop the surf beat onto land. At infragravity frequencies, the largest energy is near *f*=0.0035 Hz, which is the incident wave group period. This is strongest over the reef, but also energetic landward of the seawall. Energy is also substantial over the reef at its resonant frequency at *f*=0.0021 Hz, though this band is not as strong in the area between the beach and the house location. Energy at low frequencies *f*<0.001 Hz indicate the static setup induced by waves breaking over the reef. Energy in deep water at *f*=0.0021 Hz and *f*=0.0035 Hz is a result of reflection of the surf beat from the shore back into deep water as a free wave^21^. The fundamental basin seiche mode shows up as energy near *f*=0.006 Hz and is an artifact of the closed model domain. Consequently, energy at *f*=0.012 Hz is the first superharmonic of the basin seiche. The basin seiche shoals between the foot of the shelf slope at *x*=1,500 m and the reef crest; shoaling transfers energy of a wave to its superharmonics^44^. This results in the energy bands in Fig. 9a that connect the antinodes of the basin seiche and its superharmonics to the reef crest and gravity wave frequencies.

For the ‘no-reef' case, gravity wave energy dissipates gradually between the reef crest and the beach at *x*=3,200 m. Almost no energy in the gravity wave range exists landward of the seawall at *x*=3,314 m. At infragravity frequencies, the same basin seiches as in the ‘reef' case are present, though they contain no energy landward of the seawall. Significant energy near the wave group frequency *f*=0.0035 Hz does exist landward of the seawall; however, the amount of energy is smaller than in the real reef case. The only other infragravity energy present is at a very low frequency, representing the static setup.

**Figure 6:**
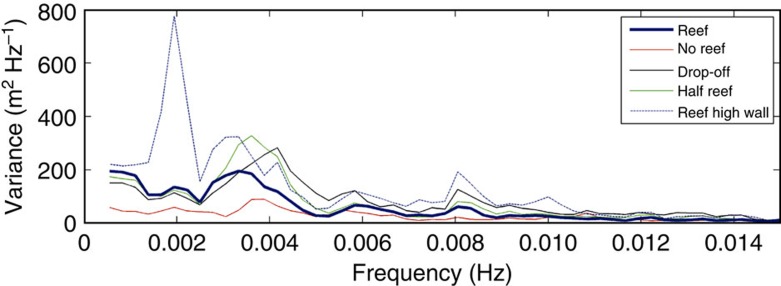
Spectral analysis showing the physics responsible for the wave in Hernani. Variance spectral density of water surface elevation from the one-dimensional BOSZ model at the location of the beach.

Figure 9 shows that between *x*=2,700 m (offshore extent of the surf zone) and *x*=3,200 m (beach), the ‘no-reef' case displays more energy than the ‘reef' scenario. However, landward of the beach, much more infragravity energy exists with the ‘reef' bathymetry. Schäffer^45^ proved that two effects control the amount of infragravity energy present at a beach: the incident bound wave groups and the time-varying migration of the gravity wave break point. Since these processes oppose each other to some extent, short surf zones experience less dissipation of infragravity energy than broad ones. Interestingly, the variance spectral density of 200 m^2^ Hz^−1^ present over a frequency range of 0.002 Hz in Fig. 6 is similar in magnitude to the computed variance spectral densities of past seismic tsunamis events over reefs in Samoa^15^ and along the shoreline of Chile^46^.

### Bore formation

van Dongeren *et al*.^23^ used laboratory experiments and an infragravity wave model to show that surf beat caused by wave groups can break and form a bore over gently sloping beaches. Battjes *et al*.^47^ formulated a dimensionless normalized bed slope parameter for infragravity waves as shown in equation (5)


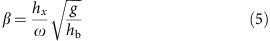


where *h*_*x*_ is the bed slope, ω is the radial frequency of the infragravity wave, *g* is the acceleration due to gravity and *h*_b_ is the depth of wave breaking. Their Fig. 2 shows that for the mild slope case of β<0.5, shoaling amplifies the surf beat and it can form a bore. For the steep slope case of β>0.5, most of the infragravity energy is reflected, so a bore does not form. In a subsequent study, van Dongeren *et al*.^48^ simulated the behaviour of surf beat over a reef and found that over a rough reef a bore does not form because bottom friction alone is sufficient to dissipate the energy of the surf beat, but over a frictionless reef a small bore forms. However, this study only considered small incident waves, on which frictional dissipation has a large influence.

**Figure 7:**
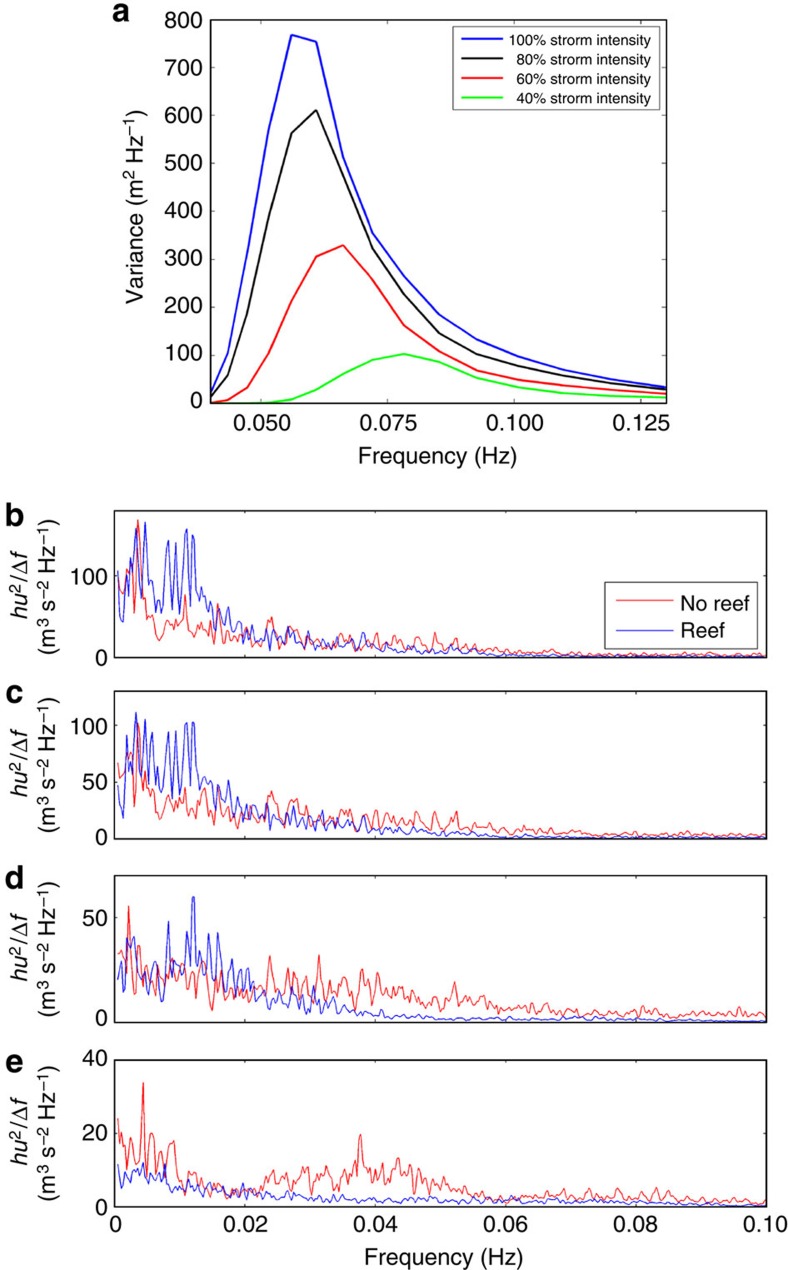
Effect of the reef on storm damage. (**a**) Wave variance spectra for various typhoon intensities output from SWAN in 150 m water depth offshore of Hernani. These spectra are used to drive the wavemaker in the BOSZ and OpenFOAM domains of Fig. 5. The 100% intensity storm is the SWAN result using measured storm track data^9^, while the others use storm track data with pressure drop and maximum wind speed reduced to the percent amount indicated. The resulting significant wave heights for decreasing storm intensity are 19.7, 17.1, 13.8 and 7.9 m, respectively. (**b**–**e**) Spectral density of *hu*^2^, which is proportional to hydrodynamic force of equation (6), from the one-dimensional BOSZ model at the location of the beach for the 100, 80, 60 and 40% intensity storms, respectively.

**Figure 8:**
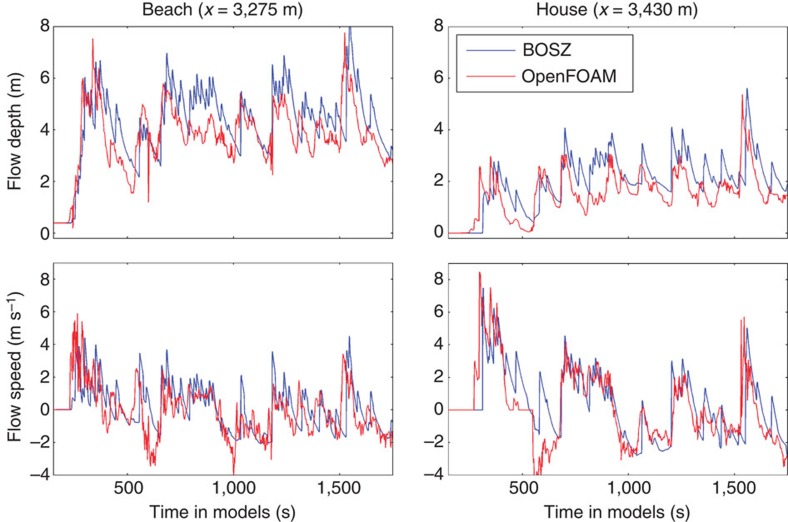
Comparison of results from OpenFOAM and one-dimensional BOSZ computations. Flow depth and speed are shown at the locations of the beach and the house of Fig. 2. The OpenFOAM and BOSZ computations use identical input wave time series and bathymetry (Fig. 5).

**Figure 9:**
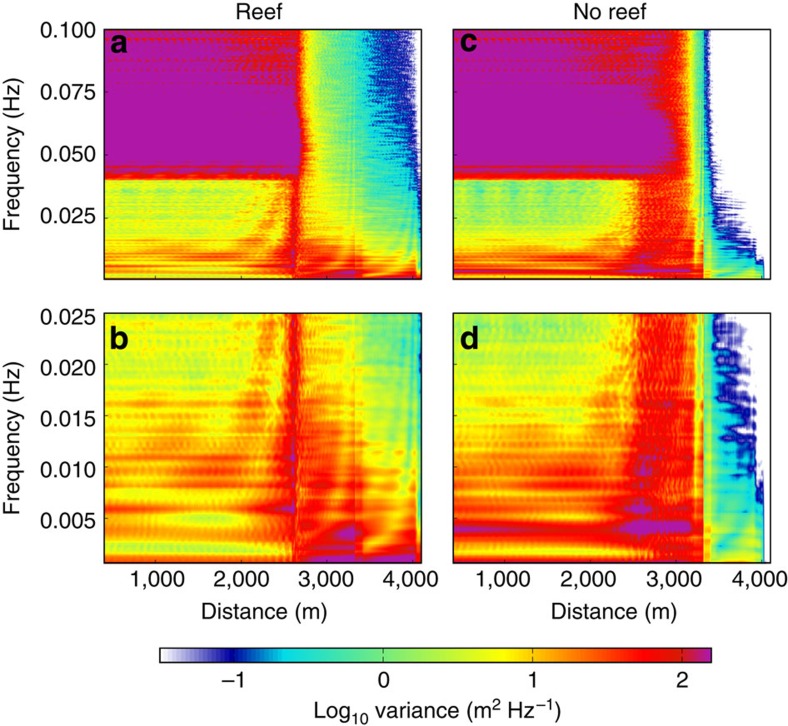
Ensemble-averaged power spectral density of water surface elevation at all points along the one-dimensional transect from BOSZ. (**a**,**b**) Plots from the real reef case and (**c**,**d**) plots from no-reef case. Panels **a** and **c** show the full frequency range, while panels **b** and **d** are zoomed in on infragravity frequencies.

**Figure 10:**
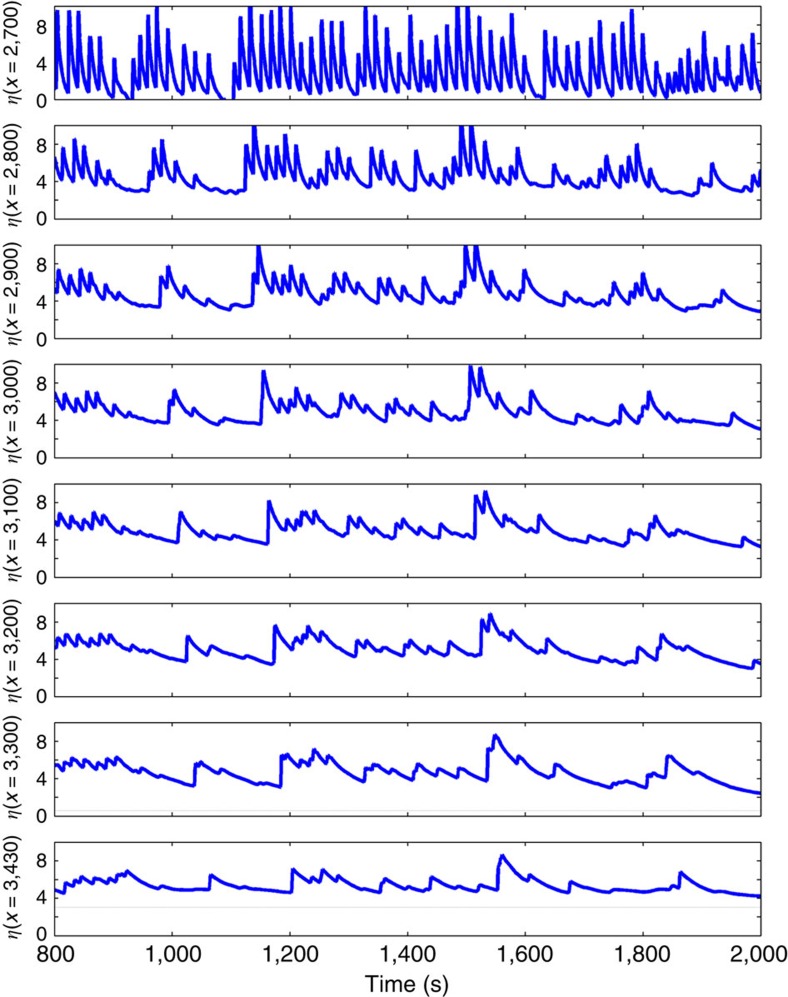
Bore formation and propagation. Time series of water level (metres above MSL) at increments from the breaking location on the reef face (*x*=2,700 m), atop the reef (*x*=2,800–3,200 m), up the beach in front of the seawall (*x*=3,000 m) and at the location of the house in Fig. 2 (*x*=3,430 m). All *x* values refer to distance along the transect of Fig. 5. At the locations on shore (*x*=3,300 and 3,430 m), the grey line indicates the elevation of the ground.

In the case of Hernani, the slope of the reef face is *h*_*x*_=0.2 and the slope of the beach is *h*_*x*_=0.017 (Fig. 5). The offshore significant wave height is 19.7 m. Assuming a wave-breaking parameter of 0.75 (ref. 43), the water depth where waves break would be approximately *h*_b_=15 m. For the wave spectrum of Typhoon Haiyan (the 100% storm of Fig. 7a), the wave group period is *T*_envelope,min_=230 s, resulting in *ω*=0.027 radians per s. This results in β=6.5 for the reef face and *β*=0.5 for the beach. Thus, bore formation is not expected over the steep reef face between the break point and the reef crest, but is possible over the beach shoreward of the reef. BOSZ computations (Fig. 10) show that at some times the surf beat rises gradually over the reef and then steepens into a bore on the beach. This is the case where the bore strikes the location of the house at *t*=1870, s. At other times, such as at *t*=1,200 s, a bore forms at the reef crest and maintains its shape across the entire reef and then up the beach. Over the reef itself, short-period gravity waves are dissipated, but the infragravity signal is not significantly affected. In the steep ‘drop-off' case (not shown), infragravity bore formation is similar, but short-period gravity waves are less damped than in the case with the reef.

Though Fig. 10 shows multiple waves striking the house over 1,200 s of BOSZ computation, it is important to note that this result was obtained with the assumption of a stationary input sea state based on the SWAN and Delft-3D outputs at 06:00 JST. At this time the sea state was still near its maximum and the still water setup had also reached its highest level (Fig. 3a). However, after 06:00 JST, SWAN computes that offshore wave heights dropped rapidly (not shown), even though the pressure- and wind-induced setup persisted. Since the pressure- and wind-driven setup lagged the offshore sea state, only a short time window existed for the surf beat to reach its most destructive form. It might be due to this short time window that residents reported only three or four infragravity waves striking the town, while Fig. 10 shows a greater number of waves.

### Drag law for hydrodynamic force

The hydrodynamic force on a building^26^ can be estimated by equation (6)


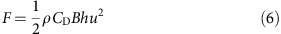


where *ρ* is the water density, *C*_D_ is an empirical drag coefficient, *B* is the width of an exposed building in the cross-flow direction, *h* is the flow depth and *u* is the flow speed.

## Additional information

**How to cite this article:** Roeber, V. & Bricker, J .D Destructive tsunami-like wave generated by surf beat over a coral reef during Typhoon Haiyan. *Nat. Commun.* 6:7854 doi: 10.1038/ncomms8854 (2015).

## Supplementary Material

Supplementary Movie 1 Animation of the water surface over the entire BOSZ model domain. The black triangle is the location of the house in Fig. 2. Grayscale indicates topography. Offshore and lateral boundary wavemakers and sponge layers are visible.

Supplementary Movie 2 Animation of flow depth and velocity vectors during the incidence of a large wave set into Hernani. The black triangle is the location of the house in Fig. 2. White lines are sea bottom elevation contours beginning at mean sea level (MSL) and continuing downward at 1 m intervals. Black lines are land elevation contours beginning at 1 m above MSL and continuing upward at 1 m intervals to 10 m above MSL (higher elevations omitted).
